# Severe Combined Immunodeficiency (SCID) and Its New Treatment Modalities

**DOI:** 10.7759/cureus.47759

**Published:** 2023-10-26

**Authors:** Akshad M Wadbudhe, Revat J Meshram, Shivangi C Tidke

**Affiliations:** 1 Department of Paediatrics, Jawaharlal Nehru Medical College, Datta Meghe Institute of Higher Education and Research, Wardha, IND

**Keywords:** immuno-chemotherapy, enzyme replacement therapy (ert), gene-replacement therapy, hematopoietic stem cell transplant, hemato-immunological biomarkers, autosomal, gene mutation, x-linked genetic diseases

## Abstract

Severe combined immunodeficiency (SCID) is a rare condition with very high mortality. SCID is mainly caused by the multiple mutations of genes affecting the entire immune cells. Children with this disease are born with an impaired immune system. The child appears healthy but the consequences of the impaired immune system lead to various secondary infections such as meningeal infections and respiratory infections further leading to consolidation, diarrhea, inflammation of skin and other systemic diseases. Severe combined immunodeficiency is also known as “bubble boy disease” or “living in the bubble” syndrome, as in early days for treatment the physicians decided to completely isolate them until they got the perfect match for the bone marrow transplantation. It is one of the pediatric emergencies and is to be treated as soon as possible. SCID involves multiple genes which leads to makes diagnosis of the disease cumbersome. In early years many infants were diagnosed almost after half a year and in severe conditions which led to the decrease in the survival rate of the children. But now due to advanced newborn screening modalities and other monitoring systems it can be diagnosed as early as within three months of age. The various treatment modalities include hematopoietic stem cell transplantation, gene therapy, enzyme replacement therapy and chemotherapy. This narrative review article describes about the severe combined immunodeficiency and its newer treatment modalities.

## Introduction and background

Severe combined immunodeficiency (SCID)

Severe combined immunodeficiency is an inherited primary immunodeficiency caused due to a defect in the hematopoietic stem cell leading to T lymphocytes and B lymphocytes depletion and abnormal function of these cells [1]. The incidence is around 1 in 58,000 live births in India for typical SCID, leaky SCID and Omenn syndrome as per the epidemiological data till 2019. There are different forms of severe combined immunodeficiency. There are defects in thymocyte maturation, production of natural killer (NK) cells, T cells and B cells along with their impaired functioning which further leads to loss in cellular and humoral immunity. If the disease is left untreated then it may even lead to death [2]. The secondary infections caused involve all the systems of the body. The neurological manifestations include meningitis, cerebral abscess and chicken pox; respiratory manifestations including pneumonia, bronchitis, lung abscess and upper respiratory tract infections; gastointestinal manifestations including diarrhea, gastritis, liver infections, pancreatitis and small intestinal infections; cardiovascular manifestations like infective endocarditis, pericarditis and vasculitis; renal manifestations like glomerulonephritis and urinary sepsis along with rheumatological and endocrinal problems [3]. The genes affected are recognized up to 75% to 80%. The treatment for severe combined immunodeficiency of these genes is definitive and are mostly treated with allogenic hematopoietic stem cell transplantation [4].

Methodology

The terms severe combined immunodeficiency, treatment modalities, primary immunodeficiencies, hematopoietic stem cell transplantation, chemotherapy, and enzyme replacement were used for the review article in PubMed. The timeline was set as 2009-till date with filters like articles, case study, surveys and other manuscripts. Around 40 articles were analyzed and sorted according to the given headings. Some of the text was also referred from Google Scholar and textbooks. The same terms were used in the Google search engine to search for any useful websites. After collection of all the data and material, all the headings and subheadings were decided. The narrative review article is written by referring to all the components collected and modified according to my knowledge and understanding.

## Review

Types

There are almost 20 different types of severe combined immunodeficiencies. Some of the commonly known types are classified according to their gene and their relation to the number of T cells, B cells, and NK cells. Table 1 shows the types of severe combined immunodeficiency [5].

**Table 1:** Types of Severe Combined Immunodeficiency SCID: Severe combined immunodeficiency; T: T lymphocytes; B: B lymphocytes; NK: Natural killer cells; IL2R: Interleukin 2 receptor; ADA: Adenosine deaminase; RAG: Recombination activating gene; IL7R: Interleukin 7 receptor; CD: Cluster differentiation; PTPRC: Protein tyrosine phosphatase type C; NHEJ: Non homologous end joining factor; PKCS: Protein kinase catalytic subunit; PRKDC: Protein kinase DNA activated catalytic subunit; JAK: Janus kinase; LAT: Linker for activation of T cells; AK: Adenylate kinase; +: high count; -: low count

	Type of Disease	T/B/NK	Gene	Hereditary
	Typical SCID:			
1)	X-linked SCID	T-/B+/NK-	IL2R gamma	X-linked
2)	Adenosine Deaminase Deficiency SCID	T-/B-/NK-	ADA	Autosomal Recessive
3)	RAG-1 AND RAG-2 Deficiency SCID	T-/B-/NK+	RAG-1 and RAG-2	Autosomal Recessive
4)	IL7R Deficiency SCID	T-/B+/NK+	IL7R alpha	Autosomal Recessive
	Other SCID:			
1)	CD3 Complex Component Deficiency SCID	T-/B+/NK+	CD3D CD3E CD247	Autosomal Recessive
2)	CD45 Deficiency SCID	T-/B+/NK+	PTPRC	Autosomal Recessive
3)	Cernunnos-XLF Deficiency SCID	T-/B-/NK+	NHEJ1	Autosomal Recessive
4)	Coronin-1A Deficiency SCID	T-/B+/NK+	CORO1A	Autosomal Recessive
5)	Artemis SCID	T-/B-/NK+	DCLRE1C	Autosomal Recessive
6)	DNA Ligase 4 Deficiency SCID	T-/B-/NK+	LIG4	Autosomal Recessive
7)	DNA-PKCS Deficiency SCID	T-/B-/NK+	PRKDC	Autosomal Recessive
8)	JAK3 Deficiency SCID	T-/B+/NK-	JAK3	Autosomal Recessive
9)	LAT Deficiency SCID	T-/B+/NK+	LAT	Autosomal Recessive
10)	Reticular Dysgenesis SCID	T-/B-/NK-	AK2	Autosomal Recessive
11)	Leaky SCID	T+/B+/NK+	Mainly RAG1 and RAG2 but others are also involved	Autosomal Recessive
12)	Omenn Syndrome	T-/B-/NK+	RAG1 and RAG2	Autosomal Recessive

Signs and symptoms 

The symptoms of severe combined immunodeficiency are mostly symptomatic and are overlapped by secondary infections. It presents in a few weeks or months of birth. First, it starts from viral infections and fungal infections affecting the respiratory tract then affecting the oral cavity due to candidal infection [6]. There are various organisms infecting the body with repeated occurring infections that are not affected by the antimicrobials and other antibiotics. Sometimes abnormal blood profiles can also be seen with low white blood cells (WBC) and platelet count [7]. The child may suffer with fever, cough, malaise, cause short-term suffocation, tonsillar infections, conjunctival infections, sore throat, seizures, dysfunction of absorption, granuloma, neoplasm, tuberculosis, lymph node swelling, stunted growth, tetany, repeated abscess, lupus, temperature intolerance, decrease in weight, muscular disorders, fetal growth retardation, glomerulonephritis, bone weakness, thyroid infection, low wound healing, amyloidosis, and genital abnormalities [8].

Screening

The screening of the fetus for the disease of severe combined immunodeficiency is done by using quantity oriented polymerase chain reaction to measure the total number of T lymphocyte receptor excision circles found in the blood plasma [9]. The thymus has created such types of DNA particles as a side product of the T lymphocyte formation. The screening can be done by the sample of the dried blood spot and is specifically done by using quantity oriented polymerase chain reaction of a stable gene for reduction of the machinery or any other error [10]. The count of T lymphocyte receptor excision circles is equivalent to the count for unique T lymphocytes formed, hence acts as the biomarker for the T lymphocyte formation. The screening tells about the change in the number of T lymphocyte formation in the thymus. America first started screening in 2008 in Wisconsin [11]. Severe combined immunodeficiency screening has made enormous changes in early diagnosing and getting results with more sensitivity and specificity. There is 99.98% specificity in epidemiological studies like cohorts for children with this type of disease [12]. The false positive rate is very low in this screening and the number of the T lymphocyte receptor excision circle can be calculated accurately [13]. Even if there is a slight chance of a false positive patient, it is cleared with follow-up T lymphocyte enumeration [14]. Other investigations for the evaluation of the disease involve white blood cell components like the total lymphocyte count, T cells and B cells, CD4 to CD8 ratio and NK cells count. The lymphocytes evaluating assays are phorbol ester and ionophore, anti-serum to CD3 and phytohemagglutinin [15]. The number of immunoglobulins like IgG, IgM, IgA and IgE can also be considered. The antibodies' activities and phagocytic functions are also tested for the diagnosis and evaluation of the disease [16]. The rare cause can be related to the autoimmune factors of the body that are to be tested [17]. The studies that involve autoimmune factors are anti-nuclear antibodies (ANA), and systemic disorders affecting some particular autoimmune antibodies (rheumatoid factor, anti-histones, anti-smith, anti-ds DNA, anti-SS-A) [18].

Treatment

The newborn child suffering from severe combined immunodeficiency would require frequent hospitalization with many tests and severe painful examinations. The patient is completely isolated, like being in a bubble. This is because there are high chances of getting an infection from the surroundings through sources such as the parents, relatives or the hospital setting itself. The cytomegalovirus is one of the most easily infecting viruses in patients with severe combined immunodeficiency. This virus could lead to years of difficulties in the child leading to disabilities and long-duration symptoms. The child needs to be isolated from overcrowded areas like playgrounds, group child care, shops and many other public places. There is no major role of nutrition and diet in this disease, but it is important for the proper growth and development of the child. The newborn with severe combined immunodeficiency is not able to sustain and absorb the given diet due to chronic diarrhoea resulting in malnutritional diseases. For maintaining nutrition intravenous diet is provided as the child is in a weak condition for any oral feed [19]. The symptomatic treatment is the first line of treatment for the disease. The child needs to be stabilized and cleared of all the secondary or opportunistic infections from the body by treatment with various classes of drugs like anti-bacterial, anti-viral, anti-fungal, anti-helminthic and other related drugs. The specific treatment for the gene mutation cause is done by the following therapy: hematopoietic stem cell transplantation (HSCT), gene therapy, enzyme replacement therapy (ERT), and chemotherapy.

Hematopoietic cell transplantation

The mortality rates for hematopoietic cell transplantation (HCT) in patients with this disease have shown differences in various studies. In retrospective studies involving multiple centers, overall survival ranged from 65% to 70%. In more recent prospective studies, the rates were higher, between 85% and 90% [20]. The most significant factor affecting mortality was the use of matched sibling donors (MSD), which had better outcomes compared to other donor categories. An in-depth analysis involving 571 non-MSD HSCT patients found that several factors influenced survival, including age, infection status at the time of HSCT, genotype, fetal thymus transplantation (FTT), and race/society [21]. Skipping a conditioning regimen (CR) has potential benefits, such as avoiding chemotherapy-related side effects and reducing the risk of graft-versus-host disease (GvHD) due to less stress on recipient epithelial cells. However, it limits the possibility of donor myeloid engraftment, mainly resulting in donor T-lymphocyte progenitor engraftment [22]. Whereas with other side, using CR with myeloablative agents generally promotes stem cell engraftment, leading to better reconstitution of T- and B-lymphocytes. Retrospective analyses found no significant difference in survival between no-CR/immunosuppression-only approaches and reduced intensity conditioning (RIC) or myeloablative conditioning (MAC) containing busulfan [23]. If there's an active infection during transplantation, using CR was associated with poorer survival outcomes. In such cases, a two-step procedure was proposed: an initial HCT without CR, followed by a second HCT with CR once the infection clears, which facilitates stem cell engraftment. For patients with RAG and DCLRE1C deficiencies, using CR makes sense due to residual NK cell function and the need to clear thymic and marrow niches for donor progenitor engraftment [24]. However, it becomes complicated in cases of DCLRE1C deficiency and other DNA repair defects, as CR has been linked to severe long-term toxicity. As a result, alternative strategies like a chemotherapy-free myeloablative approach with anti-stem cell monoclonal antibodies are being explored. Typically, HSCT with MSDs is done without CR and has survival rates exceeding 90% [25]. Some centers are now investigating reduced toxicity CR approaches for MSDs to enhance donor B-lymphocyte engraftment and avoid extended immunoglobulin replacement therapy [26].

Gene therapy 

Gene therapy is very positively effective in treating SCID patients. This process involves introducing a viral vector containing the corrected gene into the patient's own HSCs, incorporating it into their genetic makeup, and then reintroducing these treated cells into the body [27]. It has effectively cured individuals with X-linked and ADA-deficient SCID. However, initial trials faced challenges such as graft failure, and in some cases, the insertion of the vector caused mutations, leading to lymphoproliferation and leukemia, especially in X-linked SCID cases [28]. To reduce these risks, changes have been made to the design of retroviral vectors. This includes using self-inactivating gamma-retroviral vectors with modified U3 regions, as well as lenti-viral vectors. These adjustments aim to minimize the likelihood of mutations during the integration process [29]. Emerging gene editing techniques involve specific DNA cleavage enzymes that remove the faulty gene from the genome and replace it with the corrected version through the cell's natural DNA repair mechanisms. This approach allows for precise control over gene expression, making it a more biologically accurate method of genetic correction [30]. While tests in cell lines have shown promise, achieving complete correction of the genetic defect in primary HSCs has been limited. In current gene therapy protocols, achieving full correction of the genetic defect is not consistently attainable [31]. Some trials have even used low-dose chemotherapy to improve the engraftment of treated stem cells and give them a competitive advantage over untreated cells. With the potential widespread implementation of SCID screening, a chemotherapy-free approach to conventional HSCT or gene-targeted therapy could become feasible. This would eliminate concerns about long-term side effects and ensure a lasting and effective immune recovery [32].

Enzyme replacement therapy

The enzyme polyethylene glycol-conjugated adenosine deaminase (PEG-ADA) known as Revcovi, which is used in ERT, is available for both children and adults who have ADA-SCID. People with ADA-SCID lack a vital enzyme adenosine deaminase which is crucial for their immune system to function properly. In ERT, children with ADA-SCID receive regular injections of Revcovi into their muscles, which contains the missing enzyme. These injections help restore normal immune function. After initial guidance, parents can administer these injections at home, eliminating the need for medical personnel. ERT is a significant temporary solution in treating ADA-SCID patients, even if they plan to pursue a more permanent intervention like HSCT soon. The reason behind this is that short-term use of ERT quickly reduces the levels of specific toxins that build up in the body due to the absence of the ADA enzyme. These toxins lead to the destruction of white blood cells, including T cells, which is the cause of SCID. Administering ERT to infants before HSCT increases T cell counts, offering improved resistance to infections until the definitive treatment is carried out. In some cases, ERT might be extended over a longer period, spanning from months to years [33]. However, medical professionals generally consider HSCT or gene therapy as the preferred long-term solutions. The duration of ERT usage and the best time to stop may vary depending on whether the child proceeds with gene therapy or HSCT. Physicians will discuss this with parents to determine the most suitable course of action. Moreover, although not conclusively proven, it is suspected that elevated toxin levels might be the underlying cause or a contributing factor to secondary complications that can occur in ADA-SCID cases. While many ADA-SCID patients may not experience significant secondary issues, some may face challenges like liver problems, skeletal issues, skin tumors, or neurological disorders. Neurological problems can include conditions such as hearing loss, learning disabilities, attention-deficit/hyperactivity disorder (ADHD), impaired fine motor skills, involuntary rapid eye movement (nystagmus), abnormal gait, and epilepsy. Reducing toxin exposure as early as possible could potentially lower the risk or severity of these secondary complications. However, one drawback of ERT is the need for regular muscle injections, usually once or twice a week. Additionally, this treatment requires a lifelong commitment and is associated with side effects like coughing, vomiting, anemia, skin concerns, and the risk of immune system-related cancers [34]. 

Chemotherapy 

Once a proper diagnosis is confirmed, the discussion turns to the most effective treatment approach. One option is to introduce unfractionated donor HSC without using preparative chemotherapy, which helps with T-lymphocyte immune recovery [35]. However, the success of establishing thymopoiesis and the durability of T-lymphocyte function depends on the patient's phenotype and genotype. Infants with NK cell-negative SCID have better survival chances compared to those with recipient NK cells. They also have a significant presence of donor T-lymphocytes, ensuring a lasting CD4+ T-lymphocyte immunity without the need for prior chemotherapy. On the other hand, if recipient NK cells are present, it strongly suggests the need for preparative chemotherapy to facilitate the engraftment of T-lymphocyte precursors, which can support strong and long-lasting T-lymphocyte reconstitution [36]. However, the choice of the donor also matters; using an unrelated HLA-matched donor significantly increases the risk of GvHD compared to using an HLA-matched sibling donor. There are several concerns associated with using chemotherapy conditioning. Acute toxicities are commonly observed, and if an active infection is present, mortality rates rise unless a matched sibling donor is available. While durability and sustainability of thymopoiesis and B-lymphocyte function are more likely in most SCID forms following chemotherapy, especially in those with recipient NK cells, worries arise about the effects, even in short durations, of chemotherapy on young infants [37]. Currently, there are no comprehensive multicenter studies evaluating the long-term (over 20 years) immunological, overall health, or quality-of-life outcomes of HSCT in SCID. This applies to both chemotherapy preconditioning and direct donor inoculum infusion. As the majority of infants are now diagnosed during the neonatal period through newborn screening initiatives, the challenges associated with administering chemotherapy are receiving more attention [38]. While the risk of mortality from chemotherapy, especially in healthy patients with no other health issues, is low, it's not non-existent. This has led to the exploration of alternative strategies. Minimally intensive regimens that use monoclonal antibodies have effectively treated SCID, even in the presence of significant health issues [39]. In animal models, the administration of an anti-c-Kit receptor antibody, which disrupts a vital signaling pathway involved in HSC homing, adhesion, maintenance, and survival within the hematopoietic niche, has shown some benefits when given in utero, followed by transplantation on the first day of life. While these findings are promising, further research is needed before confirming actual patient benefits. Nevertheless, clinical trials using therapeutic-grade antibodies are in the planning stages [40].

## Conclusions

In conclusion, the final remarks revolve around SCID and the strategies for its treatment. The article underscores the ongoing debate regarding the optimal therapeutic approach post-diagnosis. The infusion of unfractionated donor hematopoietic stem cell inoculum minus preparative chemotherapy emerges as a topic of discussion. This technique fosters T-lymphocyte immune revival. However, the durability of T-lymphocyte function and the establishment of thymopoiesis are outcomes that fluctuate, contingent upon the phenotype and genotype factors. Notably, infants bearing NK cell-negative SCID exhibit improved survival odds in contrast to those with recipient NK cells.

The article also draws attention to the challenges associated with chemotherapy administration, especially as an increasing number of infants are diagnosed through neonatal screening initiatives. While the risk of mortality tied to chemotherapy, especially in healthy patients is low, the concerns surrounding the administration of such potent drugs to newborns have spurred the exploration of alternative therapeutic strategies. Among these approaches, minimally intensive regimens utilizing monoclonal antibodies have shown promise in treating SCID, even in cases with significant co-morbidities. However, these regimens still entail the use of low-dose chemotherapeutic agents. Unfortunately, specific treatments like alemtuzumab monotherapy or the combination of plerixafor with granulocyte-colony-stimulating factor do not seem to facilitate successful donor stem engraftment in patients. Animal models exploring the administration of an anti-C-Kit receptor antibody in utero, followed by transplantation on the first day of life, have yielded some encouraging results, enhancing donor stem cell engraftment to a certain extent.

Nevertheless, further research is necessary before establishing the true benefits for patients. Currently, plans are being developed for clinical trials employing therapeutic-grade antibodies to provide a potential path forward in SCID treatment.
